# Association of Body Mass Index With Blood Pressure Among 1.7 Million Chinese Adults

**DOI:** 10.1001/jamanetworkopen.2018.1271

**Published:** 2018-08-17

**Authors:** George C. Linderman, Jiapeng Lu, Yuan Lu, Xin Sun, Wei Xu, Khurram Nasir, Wade Schulz, Lixin Jiang, Harlan M. Krumholz

**Affiliations:** 1 Center for Outcomes Research and Evaluation, Yale–New Haven Hospital, Yale University, New Haven, Connecticut; 2 National Clinical Research Center of Cardiovascular Diseases, State Key Laboratory of Cardiovascular Disease, Fuwai Hospital, National Center for Cardiovascular Diseases, Chinese Academy of Medical Sciences and Peking Union Medical College, Beijing, People’s Republic of China; 3 Center for Prevention and Wellness Research, Baptist Health Medical Group, Miami Beach, Florida; 4 Department of Laboratory Medicine, Yale School of Medicine, New Haven, Connecticut

## Abstract

**Importance:**

Body mass index (BMI) is positively associated with blood pressure (BP); this association has critical implications for countries like China, where hypertension is highly prevalent and obesity is increasing. A greater understanding of the association between BMI and BP is required to determine its effect and develop strategies to mitigate it.

**Objective:**

To assess the heterogeneity in the association between BMI and BP across a wide variety of subgroups of the Chinese population.

**Design, Setting, and Participants:**

In this cross-sectional study, data were collected at 1 time point from 1.7 million adults (aged 35-80 years) from 141 primary health care sites (53 urban districts and 88 rural counties) from all 31 provinces in mainland China who were enrolled in the China PEACE (Patient-Centered Evaluative Assessment of Cardiac Events) Million Persons Project, conducted between September 15, 2014, and June 20, 2017. A comprehensive subgroup analysis was performed by defining more than 22 000 subgroups of individuals based on covariates, and within each subgroup, linearly regressing BMI to BP.

**Main Outcomes and Measures:**

Systolic BP was measured twice with the participant in a seated position, using an electronic BP monitor.

**Results:**

The study included 1 727 411 participants (1 027 711 women and 699 700 men; mean [SD] age, 55.7 [9.8] years). Among the study sample, the mean (SD) BMI was 24.7 (3.5), the mean (SD) systolic BP was 136.5 (20.4) mm Hg, and the mean (SD) diastolic BP was 81.1 (11.2) mm Hg. The increase of BP per unit BMI ranged from 0.8 to 1.7 mm Hg/(kg/m^2^) for 95% of the subgroups not taking antihypertensive medication. The association between BMI and BP was substantially weaker in subgroups of patients taking antihypertensive medication compared with those who were untreated. In untreated subgroups, 95% of the coefficients varied by less than 1 mm Hg/(kg/m^2^).

**Conclusions and Relevance:**

The association between BMI and BP is positive across tens of thousands of individuals in population subgroups, and, if causal, given its magnitude, would have significant implications for public health.

## Key Points

**Question:**

How does increasing body mass index affect the prevalence of hypertension and cardiovascular health in different groups of people in China?

**Findings:**

In this population-based cross-sectional study, the association between body mass index and blood pressure was consistent and positive across more than 20 000 subgroups of 1.7 million Chinese adults and was only substantially affected by treatment with antihypertensive medication.

**Meaning:**

Trends in increasing body mass index may lead to increases in blood pressure and cardiovascular events, suggesting the need for measures of better prevention of this increase, as well as mitigation of hypertension with treatment and lifestyle measures.

## Teaser

This population-based cross-sectional study of 1.7 million Chinese adults assesses the association between body mass index (BMI), blood pressure, and hypertension across subgroups of age, sex, income, race, smoking, stroke, and more.

## Introduction

Body mass index (BMI) is positively associated with both systolic blood pressure (SBP) and diastolic blood pressure (DBP).^1,2,3,4,5^ Weight loss significantly reduces blood pressure (BP),^6,7,8^ suggesting that BMI is not merely a marker of factors associated with high BP but is causally associated.^9,10,11^ One implication of this association is that elevated BP, a major cardiovascular disease risk factor, will become an even more dominant health challenge as BMI continues to increase.

The association between BMI and BP has important implications in countries such as China, where hypertension is already common and the prevalence of obesity is projected to more than triple in men (from 4.0% to 12.3%) and more than double in women (from 5.2% to 10.8%) by 2025, as compared with 2010.^12^ To understand this association and the strategies to mitigate it requires a greater understanding of the nature of the association of BMI with BP and any heterogeneity in the association across diverse subgroups. In particular, it is important to go beyond simple stratifications of a few demographic characteristics and investigate subgroups defined by their age, sex, race/ethnicity, geography, occupation, and other pertinent characteristics. To our knowledge, studies have not addressed how consistent the association between BMI and BP is across such a diversity of groups.

Accordingly, we used data from a study of 1.7 million participants from China-PEACE (Patient-Centered Evaluative Assessment of Cardiac Events) Million Persons Project,^13^ a nationwide Chinese screening project, to systematically assess the nature of the association between BMI and BP and how it varies across a wide variety of subgroups of the population. Such knowledge could suggest ways to target interventions, provide insight into mechanisms, and project future health care needs. This issue is particularly germane to the fifth of the world’s population in China but also has general relevance because many countries experience trends of increasing weight and face challenges with hypertension.

## Methods

### Participants and Study Design

The design and conduct of the China-PEACE Million Persons Project have been previously described in detail.^13^ In brief, the China-PEACE Million Persons Project is a national, population-based screening project funded by the Chinese government to identify individuals at high risk of cardiovascular disease and to collect research-grade data and biospecimens from this population. From September 15, 2014, to June 20, 2017, we used a convenience sampling strategy to select 141 primary health care sites (88 rural counties and 53 urban districts) from all 31 provinces in mainland China. In each site, 5 towns or subdistricts were chosen according to their size and population stability. In each town or subdistrict, potential participants were invited by local staff via extensive publicity campaigns on television and in the newspaper. Participants were enrolled if they were between the ages of 35 and 80 years and could confirm their residence in a selected region. On enrollment, participants were screened for high risk of cardiovascular disease using measurements including weight, height, BP, serum lipid levels, and a questionnaire evaluating general health status. An interviewer measured each participant’s BP twice with the participant in a seated position after 5 minutes of rest, using an electronic BP monitor (Omron HEM-7430; Omron Corp) on the right upper arm, with a 1-minute delay between measurements. If the difference between the 2 SBP measurements exceeded 10 mm Hg, a third measurement was taken, and the mean of the last 2 measurements was used. The participant’s weight and height without shoes and wearing light clothing were then measured by trained technicians. Body mass index was then calculated as weight in kilograms divided by height in meters squared. In addition to physical measurements, sociodemographic data and data on basic medical history were collected from standardized in-person interviews by trained medical staff. At present, data have been collected from 1 822 229 participants aged 35 to 80 years at 1 time point. The data collection period covered only 39 months, precluding a trend analysis. We excluded participants with missing or obviously erroneous data or those belonging to ethnic groups with fewer than 5000 participants, resulting in a group of 1 727 411 individuals. The central ethics committee at the China National Center for Cardiovascular Disease approved this project, and the institutional review board at Yale University reviewed the study and waived the requirement for informed consent. The study was reported in accordance with the Strengthening the Reporting of Observational studies in Epidemiology (STROBE) reporting guideline.

### Statistical Analysis

All statistical analyses were conducted using R, version 3.3 (The R Foundation for Statistical Computing). The proportion of individuals with stage 1 hypertension (SBP ≥140 mm Hg or DBP ≥90 mm Hg) and stage 2 hypertension (SBP ≥160 mm Hg or DBP ≥100 mm Hg), as classified by JNC 7 (the Seventh Report of the Joint National Committee on Prevention, Detection, Evaluation, and Treatment of High Blood Pressure),^14^ are reported. The association between BMI and BP was analyzed for subgroups of greater than 5000 patients, defined by sex, age, household income, occupation, race/ethnicity, marital status, “Hukou” status (urban vs rural vs unified residence status), province, educational level, currently smoking, history of stroke, and antihypertensive treatment status. The smoothed conditional mean of BMI given BP for these subgroups was fit with an unadjusted generative additive model to visually explore the linearity of the association between BMI and BP. Generative additive models were chosen owing to their scalability; while local regression methods (ie, Loess) can also fit nonlinear curves, they do not scale well beyond several thousand points.

Separately, the coefficient of BMI when linearly regressed to SBP was reported as the increase of SBP per 1 unit of BMI. An adjusted increase of SBP per 1 unit of BMI was computed by linear regression, including the following covariates in the regression model: sex, age, household income, occupation, race/ethnicity, marital status, Hukou status (urban vs rural vs unified residence status), province, educational level, currently smoking, history of stroke, and antihypertensive treatment status. All these variables are categorical except age, which was not discretized when added as a covariate. When computing an adjusted linear regression model for a subgroup, we excluded the variable defining that subgroup from the model. Adjusted and unadjusted increases in DBP per 1 unit of BMI were also similarly computed.

Because the distribution of SBP is expected to be different between patients taking antihypertensive medications and those not taking antihypertensive medications, we also compared the association of BMI with BP between these 2 groups after matching their distribution of SBP. Specifically, we used a histogram-based density estimation (100 equal-width bins) to estimate the distribution of SBP in both groups, and we subsampled the group of patients not taking medication such that the distribution of SBP in the resulting group would match that of patients taking antihypertensive medications. The association between BMI and BP was then compared between the group of patients taking medications and the matched subgroup of patients not taking medications.

To further explore whether and how the association between BMI and BP varies across population subgroups, we calculated the coefficient of BMI when regressed to SBP (ie, the increase in SBP [mm Hg] per 1 kg/m^2^ of BMI) for subgroups defined by all possible combinations of the covariates. In this comprehensive subgroup analysis, we defined subgroups by all possible combinations of up to 6 of 10 selected characteristics: age (35-50, 51-60, 61-70, and 71-80 years), sex (men or women), ethnicity (Han, Mongol, Hui, Tibet, Uygher, Miao, Yi, Zhuang, Korean, Man, Dong, and Tujia), Hukou status (urban vs rural vs unified residential status), marital status (married or unmarried), occupation (farmer, worker, administrator, administrative clerk, technician, business, business owner, military, other, and retired), annual household income (<¥10 000 [equivalent to $1452], ¥10 000-¥50 000 [$1452-$7259], and ≥¥50 000 [$7259]), educational level (illiterate, less than primary school, homeschool, elementary school, middle school, high school, vocational school, associate degree, bachelor degree, master’s degree, doctoral degree, and no answer), antihypertension treatment status (treated vs untreated), and province. For example, a possible subgroup defined by age, sex, and treatment status could be women 51 to 60 years of age who are not taking antihypertensive medication. We retained subgroups of more than 10 000 people, calculated the linear regression coefficient and Spearman rank correlation coefficient of BMI to SBP and to DBP, and plotted the distribution of these coefficients as histograms.

All confidence intervals correspond to 95% CIs, as computed from the SE. The data that support the findings of this study are available from L.J. upon reasonable request and funding for deidentification of protected health information in the study.

## Results

### BMI and BP

Our analysis includes 1 727 411 participants (1 027 711 women and 699 700 men), with a mean (SD) age of 55.7 (9.8) years. Among the study sample, the mean (SD) BMI was 24.7 (3.5), the mean (SD) SBP was 136.5 (20.4) mm Hg, and the mean (SD) DBP was 81.1 (11.2) mm Hg (eFigure 1 in the Supplement). The prevalence of hypertension (SBP ≥140 mm Hg or DBP ≥90 mm Hg) was 41.6% (n = 718 714), and the prevalence of stage 2 hypertension (SBP ≥160 mm Hg or DBP ≥100 mm Hg) was 15.3% (n = 263 695). Furthermore, 36 201 participants (2.1%) were underweight (BMI <18.5), 952 471 participants (55.1%) were normal weight (BMI 18.5-24.9), 622 207 participants (36.0%) were overweight (BMI 25.0-29.9), and 116 532 participants (6.7%) were obese (BMI ≥30), as defined by the World Health Organization international classification.^15^

### Association Between BMI and BP Overall and in Population Subgroups

**Figure 1.**
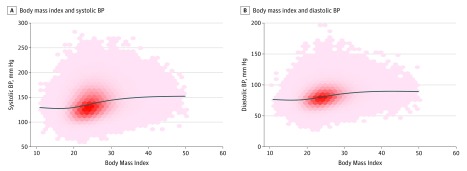
Scatterplots of Body Mass Index Against Blood Pressure (BP) A, Body mass index (calculated as weight in kilograms divided by height in meters squared) and systolic BP. B, Body mass index and diastolic BP. Darker red denotes higher density. The black line is a smoothed condition mean, denoting the mean systolic or diastolic BP at a given body mass index.

The association between BP and BMI in the overall sample is positive and linear from a BMI of 18.5 to 30.0 (Figure 1), with an unadjusted increase in SBP per unit BMI of 1.37 (95% CI, 1.35-1.93) mm Hg/(kg/m^2^), an adjusted increase in SBP per unit BMI of 1.15 (95% CI, 1.13-1.17) mm Hg/(kg/m^2^), an unadjusted increase in DBP per unit BMI of 0.85 (95% CI, 0.84-0.86) mm Hg/(kg/m^2^), and an adjusted increase in DBP per unit BMI of 0.75 (95% CI, 0.74-0.76) mm Hg/(kg/m^2^).

**Figure 2.**
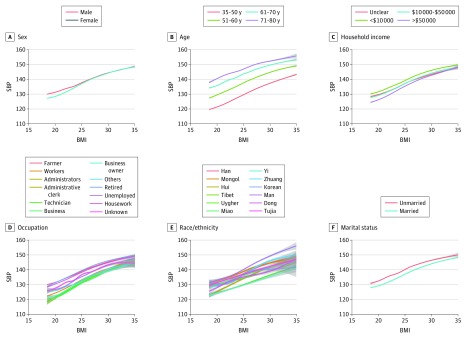
Smoothed Conditional Mean of Systolic Blood Pressure (SBP) Given Body Mass Index (BMI) for Sex, Age, Income, Occupation, Race/Ethnicity, and Marital Status The smoothed conditional mean was fitted with an unadjusted generative additive model. Body mass index is calculated as weight in kilograms divided by height in meters squared.

**Figure 3.**
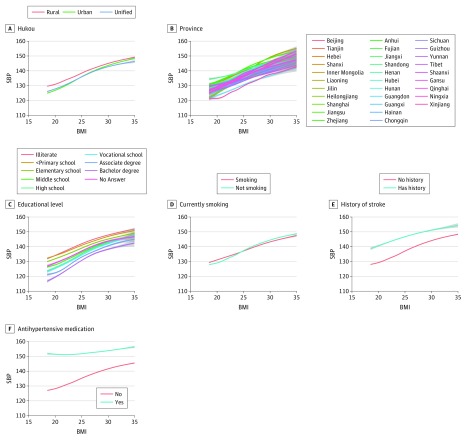
Smoothed Conditional Mean of Systolic Blood Pressure (SBP) Given Body Mass Index (BMI) for Region, Province, Educational Level, Smoking Status, Stroke History, and Antihypertensive Medication Use The smoothed conditional mean was fitted with an unadjusted generative additive model. Body mass index is calculated as weight in kilograms divided by height in meters squared.

The association of BMI with SBP (Figure 2 and Figure 3) and DBP (eFigure 2 in the Supplement) was consistently positive across 86 subgroups defined by sociodemographic variables and was nearly linear, with little variation in its shape. The positive, nearly linear association between BMI and BP across sex, age, household income, occupation, race/ethnicity, marital status, Hukou status, province, educational level, smoking status, and history of stroke is qualitatively similar (eTable 1 in the Supplement). Furthermore, the estimated unadjusted increase in SBP per unit BMI is between 1.0 and 1.9 mm Hg/(kg/m^2^) for nearly all subgroups. The only 2 exceptions are individuals from the province of Tibet, with an unadjusted increase in SBP per unit BMI of 0.56 (95% CI, 0.44-0.68) mm Hg/(kg/m^2^), and individuals taking antihypertensive medications, with an unadjusted increase in SBP per unit BMI of 0.34 (95% CI, 0.29-0.39) mm Hg/(kg/m^2^).

**Figure 4.**
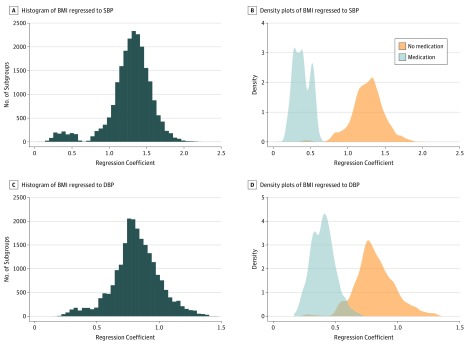
Distributions of the Coefficients of Body Mass Index (BMI) When Regressed to Systolic Blood Pressure (SBP) or Diastolic Blood Pressure (DBP) for Various Population Subgroups A, Histogram of the coefficients of BMI when regressed to SBP for subgroups defined by combinations of covariates. B, Density plots of the coefficients of BMI when regressed to SBP for subgroups defined by combinations of covariates. C, Histogram of the coefficients of BMI when regressed to DBP for subgroups defined by combinations of covariates. D, Density plots of the coefficients of BMI when regressed to DBP for subgroups defined by combinations of covariates.

The distribution of regression coefficients of BMI to SBP across all 22 333 subgroups reveals 2 modes, each corresponding to subgroups defined by treated patients and untreated patients (Figure 4). A similar distribution is also observed for the Spearman correlation between BMI and BP for each subgroup (eFigure 3 in the Supplement). The same 2 modes are also observed for regression coefficients of BMI to DBP. The association between BMI and BP in subgroups in which individuals were taking antihypertensive medications (95% of the coefficients ranging from 0.2 to 0.6 mm Hg/[kg/m^2^]) is substantially smaller compared with subgroups in which individuals were not taking antihypertensive medications (95% of the coefficients ranging from 0.8 to 1.7 mm Hg/[kg/m^2^]). This difference is also evident when comparing individuals taking medications with a matching group of individuals not taking medications (eFigure 4 in the Supplement).

## Discussion

This study provides a detailed description of the association between BMI and BP in a national population-based study in China, which has important implications for the future of cardiovascular risk. The association between BMI and BP is robust; despite dramatic differences in their individual BP distributions, the association is similar across tens of thousands of subgroups, with implications for the primary prevention of hypertension. In nearly all subgroups of individuals taking antihypertensive medications, however, this association is attenuated, indicating, as expected, that treatment mitigates the association of BMI with BP. Given these results, China and countries in a similar position should not only focus on preventing the increase of BMI but also improve diagnosis and treatment rates of hypertension to mitigate this emerging health threat.

Our study extends the literature in several ways. First, we identified the association of increasing BMI with BP in a large, well-stratified Chinese population. Second, the unprecedented number of individuals in our study allows us to study the association between BMI and BP in more than 20 000 subgroups defined by combinations of covariates. In 95% of the subgroups not taking antihypertensive medication, the coefficients varied by less than 1 mm Hg/(kg/m^2^), demonstrating the stability of this association to an extent never previously explored, to our knowledge. Furthermore, we extended recent trends of increasing BMI in China (eFigure 5 in the Supplement) to project mean BMI in 2025, and assuming the association between BMI and BP remains the same, we estimated the number of strokes and ischemic heart disease burden in 2025 attributable to the associated increase in BP (eAppendices 1 and 2 in the Supplement). Assuming mean BMI in men and women increases from 24.9 and 24.3 in 2014 to 27.8 and 25.3 in 2025, the resulting increase in BP could potentially lead to more than 300 000 cases of stroke (eTable 2 in the Supplement) and 350 000 cases of ischemic heart disease (eTable 3 in the Supplement) by 2025, highlighting the disease burden attributable to the association of BMI with BP. This estimate assumes that the association of increasing BMI with BP will persist, whether as a marker or mediator, as will the current patterns of treatment. It is a rough estimate but indicates the possibility that China will be facing an even larger challenge with hypertension in the future.

China and other countries experiencing an epidemiologic transition have 2 paths to mitigate the increasing threat to cardiovascular health posed by increasing BMI. The first is to slow the increase in BMI through public health interventions, and the second is through better treatment of hypertension. Although many public health strategies have not yet been successful in slowing the increase of BMI in adults,^16^ increasing the rates of treatment of hypertension is an attainable goal. However, a recent study based on the China PEACE Million Persons Project reported that, among people with hypertension, the rate of awareness of their hypertension was 36.0%, the rate of treatment of their hypertension was 22.9%, and the rate of control of their hypertension was 5.7%.^17^ Moreover, many primary care clinics lack antihypertensive medications even though they are identified by the government as essential medications.^18^ The implication is that better antihypertensive treatment can play a crucial role in mitigating the health effects of increasing BMI in China and that efforts should be taken to promote the use of effective antihypertensive medication among those with hypertension. In particular, the primary care and insurance systems in China should be strengthened so as to reduce the barrier for antihypertensive treatment.

Our analysis of the general association between BMI and BP is generally consistent with previous literature; for every 1-kg/m^2^ increase in BMI, our analysis shows a 1.3–mm Hg increase for men and a 1.4–mm Hg increase for women, compared with a 1.4–mm Hg increase for men and a 1.2–mm Hg increase for women in a Western study^19^ and a 1.7–mm Hg increase for men and a 1.4–mm Hg increase for women in a Chinese study.^20^ Furthermore, our finding that antihypertensive treatment mitigates the association of BMI with SBP is consistent with a previous study of 500 000 Chinese individuals from 10 locales, in which the association between BMI and SBP was found to be only one-third as strong in participants receiving antihypertensive medication.^20^

### Limitations

Our study has several limitations. First, our study is based on cross-sectional data; therefore, unlike studies that have shown that changes in BMI are independently associated with the incidence of hypertension,^1,21,22^ we cannot establish a causal association between BMI and BP. Nevertheless, based on prior literature, and especially data on the association of weight loss with BP, a causal association seems quite plausible. Second, our comparison of the strength of association between BMI and BP is based on comparing linear regression coefficients, which assumes a linear association. Visualization of the association as fit by generative additive models suggests that the association is fairly linear, and for the subgroups that we did not visualize, we validated our results using the Spearman coefficient, which assumes only a monotonic association. Third, we cannot determine if BMI is independently associated with BP, or if it is simply a surrogate for another variable. Many factors could be the modifiers of the association between BMI and BP. For some people, an increased BMI may be a result of diet or activity, a balance between diet and activity, or the result of a genetic predisposition combined with some proportion of contribution by diet and/or activity. Taking advantage of the large sample size of our study, we were able to show that the association of BMI with BP was robust and that the coefficients were similar across subgroups. Although our study cannot prove causation, as is true with observational, epidemiologic studies, it lays a foundation for projections and for testing the outcome of interventions in a country in the middle of an epidemiologic transition.

## Conclusions

The association between BP and BMI is positive in the general population and in tens of thousands of subgroups, suggesting that the trend of increasing BMI will be associated with the prevalence of hypertension across nearly all segments of the population. Treatment of BP alters this association and could play a critical role in mitigating the public health effect of increasing BMI, to complement public health measures. The estimates of the potential association of BMI with stroke and ischemic heart disease, which are based on the current pattern of treatment in this population, should signal the need for addressing the future increase in the prevalence of hypertension as a national priority in China and other countries experiencing a similar epidemiologic transition. Unless China markedly slows the increase in BMI and/or improves its rates of treatment and control of hypertension, the association of hypertension with cardiovascular risk is likely to cause hundreds of thousands more myocardial infarctions and strokes as a result of projected increases in BMI and its association with BP.

## Notes

Supplement. **eFigure 1.** Distributions of a) Systolic Blood Pressure, b) Diastolic Blood Pressure, and c) Body Mass Index (BMI) in the Study Population **eFigure 2.** Smoothed Conditional Mean of Diastolic Blood Pressure (DBP, mmHg) Given Body Mass Index (BMI, kg/m^2^), as Fit With an Unadjusted Generative Additive Model, for Various Sociodemographic Subgroups, Each With at Least 5,000 Individuals **eFigure 3.** Histograms and Density Plots of the Spearman’s Rank Correlation Coefficient of Body Mass Index With Systolic Blood Pressure (Panels a and b) or With Diastolic Blood Pressure (Panels c and d) for Every Subgroup Defined by Combinations of Covariates **eFigure 4.** Distribution of Systolic Blood Pressure (SBP) for Individuals on Medication Before and After Matching (Panels a and c), and Smoothed Conditional Mean of SBP Given Body Mass Index (BMI) Before and After Matching (Panels b and d) **eFigure 5.** BMI Estimates (Red) Extended to the Year 2030 (Blue) Using a Third Order Polynomial **eTable 1.** Characteristics of the Study Population and the Increase in Blood Pressure (mmHg) per 1 kg/m^2^ Body Mass Index for Each Subgroup **eTable 2.** Predicted Increase in Body Mass Index and Attributable Increase in Systolic Blood Pressure by 2025, Population Attributable Fraction (PAF), and Estimate for Strokes That Can Be Attributed to the Increase in Body Mass Index in Men **eTable 3.** Predicted Increase in Body Mass Index and Attributable Increase in Systolic Blood Pressure by 2025, Population Attributable Fraction (PAF), and Estimate for Ischemic Heart Disease (IHD) That Can Be Attributed to the Increase in Body Mass Index in Men **eAppendix 1.** Projected Rates of Stroke and Ischemic Heart Disease Attributable to Increasing BMI **eAppendix 2.** Calculation of Population Attributable Fraction **eReferences**

## References

[1] Drøyvold WB, Midthjell K, Nilsen TI, Holmen J Change in body mass index and its impact on blood pressure: a prospective population study. Int J Obes (Lond). 2005;29(6):-. doi:10.1038/sj.ijo.0802944 15809666

[2] Gelber RP, Gaziano JM, Manson JE, Buring JE, Sesso HD A prospective study of body mass index and the risk of developing hypertension in men. Am J Hypertens. 2007;20(4):370-377. doi:10.1016/j.amjhyper.2006.10.011 17386342 PMC1920107

[3] Shuger SL, Sui X, Church TS, Meriwether RA, Blair SN Body mass index as a predictor of hypertension incidence among initially healthy normotensive women. Am J Hypertens. 2008;21(6):613-619. doi:10.1038/ajh.2008.169 18437123 PMC3410431

[4] Mokdad AH, Ford ES, Bowman BA, Prevalence of obesity, diabetes, and obesity-related health risk factors, 2001. JAMA. 2003;289(1):76-79. doi:10.1001/jama.289.1.76 12503980

[5] Hubert HB, Feinleib M, McNamara PM, Castelli WP Obesity as an independent risk factor for cardiovascular disease: a 26-year follow-up of participants in the Framingham Heart Study. Circulation. 1983;67(5):968-977. doi:10.1161/01.CIR.67.5.968 6219830

[6] Neter JE, Stam BE, Kok FJ, Grobbee DE, Geleijnse JM Influence of weight reduction on blood pressure: a meta-analysis of randomized controlled trials. Hypertension. 2003;42(5):878-884. doi:10.1161/01.HYP.0000094221.86888.AE 12975389

[7] Harsha DW, Bray GA Weight loss and blood pressure control (pro). Hypertension. 2008;51(6):1420-1425. doi:10.1161/HYPERTENSIONAHA.107.094011 18474829

[8] Stevens VJ, Obarzanek E, Cook NR, ; Trials for the Hypertension Prevention Research Group Long-term weight loss and changes in blood pressure: results of the Trials of Hypertension Prevention, phase II. Ann Intern Med. 2001;134(1):1-11. doi:10.7326/0003-4819-134-1-200101020-00007 11187414

[9] Rahmouni K, Correia ML, Haynes WG, Mark AL Obesity-associated hypertension: new insights into mechanisms. Hypertension. 2005;45(1):9-14. doi:10.1161/01.HYP.0000151325.83008.b4 15583075

[10] Rahmouni K Obesity-associated hypertension: recent progress in deciphering the pathogenesis. Hypertension. 2014;64(2):215-221. doi:10.1161/HYPERTENSIONAHA.114.00920 24821943 PMC4184930

[11] Sowers JR Obesity as a cardiovascular risk factor. Am J Med. 2003;115(suppl 8A):37S-41S. doi:10.1016/j.amjmed.2003.08.012 14678864

[12] Collaboration NCDRF; NCD Risk Factor Collaboration (NCD-RisC) Trends in adult body-mass index in 200 countries from 1975 to 2014: a pooled analysis of 1698 population-based measurement studies with 19.2 million participants. Lancet. 2016;387(10026):1377-1396. doi:10.1016/S0140-6736(16)30054-X 27115820 PMC7615134

[13] Lu J, Xuan S, Downing NS, Protocol for the China PEACE (Patient-centered Evaluative Assessment of Cardiac Events) Million Persons Project pilot. BMJ Open. 2016;6(1):e010200. doi:10.1136/bmjopen-2015-010200 26729395 PMC4716208

[14] Chobanian AV, Bakris GL, Black HR, ; National Heart, Lung, and Blood Institute Joint National Committee on Prevention, Detection, Evaluation, and Treatment of High Blood Pressure; National High Blood Pressure Education Program Coordinating Committee The Seventh Report of the Joint National Committee on Prevention, Detection, Evaluation, and Treatment of High Blood Pressure: the JNC 7 report. JAMA. 2003;289(19):2560-2572. doi:10.1001/jama.289.19.2560 12748199

[15] Consultation WHOE; WHO Expert Consultation Appropriate body-mass index for Asian populations and its implications for policy and intervention strategies. Lancet. 2004;363(9403):157-163. doi:10.1016/S0140-6736(03)15268-3 14726171

[16] Walls HL, Peeters A, Proietto J, McNeil JJ Public health campaigns and obesity—a critique. BMC Public Health. 2011;11:136. doi:10.1186/1471-2458-11-136 21352562 PMC3056747

[17] Lu J, Lu Y, Wang X, Prevalence, awareness, treatment, and control of hypertension in China: data from 1.7 million adults in a population-based screening study (China PEACE Million Persons Project). Lancet. 2017;390(10112):2549-2558. doi:10.1016/S0140-6736(17)32478-9 29102084

[18] Su M, Zhang Q, Bai X, Availability, cost, and prescription patterns of antihypertensive medications in primary health care in China: a nationwide cross-sectional survey. Lancet. 2017;390(10112):2559-2568. doi:10.1016/S0140-6736(17)32476-5 29102087

[19] Whitlock G, Lewington S, Sherliker P, ; Prospective Studies Collaboration Body-mass index and cause-specific mortality in 900 000 adults: collaborative analyses of 57 prospective studies. Lancet. 2009;373(9669):1083-1096. doi:10.1016/S0140-6736(09)60318-4 19299006 PMC2662372

[20] Chen Z, Smith M, Du H, ; China Kadoorie Biobank Collaborative Group Blood pressure in relation to general and central adiposity among 500 000 adult Chinese men and women. Int J Epidemiol. 2015;44(4):1305-1319. doi:10.1093/ije/dyv012 25747585 PMC4588860

[21] Ren Q, Su C, Wang H, Wang Z, Du W, Zhang B Change in body mass index and its impact on incidence of hypertension in 18-65-year-old Chinese adults. Int J Environ Res Public Health. 2016;13(3):257. doi:10.3390/ijerph13030257 26927144 PMC4808920

[22] Yang G, Shu XO, Gao YT, Zhang X, Li H, Zheng W Impacts of weight change on prehypertension in middle-aged and elderly women. Int J Obes (Lond). 2007;31(12):1818-1825. doi:10.1038/sj.ijo.0803680 17653069

