# Supplementary material for: Association of Body Mass Index With Blood Pressure Among 1.7 Million Chinese Adults
Source: JAMA Netw Open. 2018 Aug 17;1(4):e181271. doi: 10.1001/jamanetworkopen.2018.1271 (PMC6324286; doi:10.1001/jamanetworkopen.2018.1271)
Supplement: Supplement. — eFigure 1. Distributions of a) Systolic Blood Pressure, b) Diastolic Blood Pressure, and c) Body Mass Index (BMI) in the Study Population eFigure 2. Smoothed Conditional Mean of Diastolic Blood Pressure (DBP, mmHg) Given Body Mass Index (BMI, kg/m2), as Fit With an Unadjusted Generative Additive Model, for Various Sociodemographic Subgroups, Each With at Least 5,000 Individuals eFigure 3. Histograms and Density Plots of the Spearman’s Rank Correlation Coefficient of Body Mass Index With Systolic Blood Pressure (Panels a and b) or With Diastolic Blood Pressure (Panels c and d) for Every Subgroup Defined by Combinations of Covariates eFigure 4. Distribution of Systolic Blood Pressure (SBP) for Individuals on Medication Before and After Matching (Panels a and c), and Smoothed Conditional Mean of SBP Given Body Mass Index (BMI) Before and After Matching (Panels b and d) eFigure 5. BMI Estimates (Red) Extended to the Year 2030 (Blue) Using a Third Order Polynomial eTable 1. Characteristics of the Study Population and the Increase in Blood Pressure (mmHg) per 1 kg/m2 Body Mass Index for Each Subgroup eTable 2. Predicted Increase in Body Mass Index and Attributable Increase in Systolic Blood Pressure by 2025, Population Attributable Fraction (PAF), and Estimate for Strokes That Can Be Attributed to the Increase in Body Mass Index in Men eTable 3. Predicted Increase in Body Mass Index and Attributable Increase in Systolic Blood Pressure by 2025, Population Attributable Fraction (PAF), and Estimate for Ischemic Heart Disease (IHD) That Can Be Attributed to the Increase in Body Mass Index in Men eAppendix 1. Projected Rates of Stroke and Ischemic Heart Disease Attributable to Increasing BMI eAppendix 2. Calculation of Population Attributable Fraction eReferences [file jamanetwopen-1-e181271-s001.pdf]

Supplementary Online Content

Linderman GC, Lu J, Lu Y, et al. Association of body mass index with blood pressure among 1.7 million Chinese adults. *JAMA Netw Open*. 2018;1(4):e181271. doi:10.1001/jamanetworkopen.2018.1271

eFigure 1. Distributions of a) Systolic Blood Pressure, b) Diastolic Blood Pressure, and c) Body Mass Index (BMI) in the Study Population
eFigure 2. Smoothed Conditional Mean of Diastolic Blood Pressure (DBP, mmHg) Given Body Mass Index (BMI, kg/m^2), as Fit With an Unadjusted Generative Additive Model, for Various Sociodemographic Subgroups, Each With at Least 5,000 Individuals
eFigure 3. Histograms and Density Plots of the Spearman's Rank Correlation Coefficient of Body Mass Index With Systolic Blood Pressure (Panels a and b) or With Diastolic Blood Pressure (Panels c and d) for Every Subgroup Defined by Combinations of Covariates
eFigure 4. Distribution of Systolic Blood Pressure (SBP) for Individuals on Medication Before and After Matching (Panels a and c), and Smoothed Conditional Mean of SBP Given Body Mass Index (BMI) Before and After Matching (Panels b and d)
eFigure 5. BMI Estimates (Red) Extended to the Year 2030 (Blue) Using a Third Order Polynomial
eTable 1. Characteristics of the Study Population and the Increase in Blood Pressure (mmHg) per 1 kg/m^2 Body Mass Index for Each Subgroup
eTable 2. Predicted Increase in Body Mass Index and Attributable Increase in Systolic Blood Pressure by 2025, Population Attributable Fraction (PAF), and Estimate for Strokes That Can Be Attributed to the Increase in Body Mass Index in Men
eTable 3. Predicted Increase in Body Mass Index and Attributable Increase in Systolic Blood Pressure by 2025, Population Attributable Fraction (PAF), and Estimate for Ischemic Heart Disease (IHD) That Can Be Attributed to the Increase in Body Mass Index in Men
eAppendix 1. Projected Rates of Stroke and Ischemic Heart Disease Attributable to Increasing BMI
eAppendix 2. Calculation of Population Attributable Fraction
eReferences

This supplementary material has been provided by the authors to give readers additional information about their work.

© 2018 Linderman GC et al. *JAMA Network Open.*

eFigure 1. Distributions of a) systolic blood pressure, b) diastolic blood pressure, and c) body mass index (BMI) in the study population

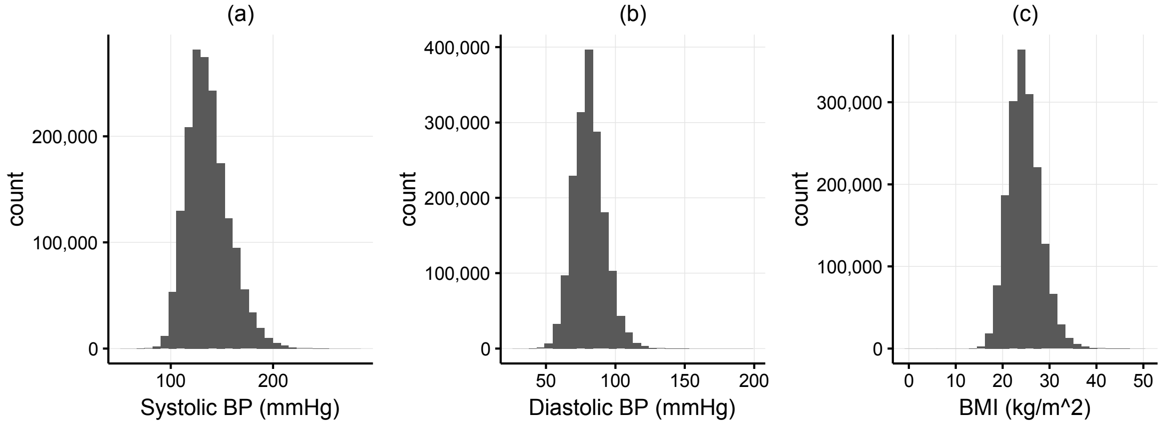

eFigure 2. Smoothed conditional mean of diastolic blood pressure (DBP, mmHg) given body mass index (BMI, kg/m^2), as fit with an unadjusted generative additive model, for various sociodemographic subgroups, each with at least 5,000 individuals

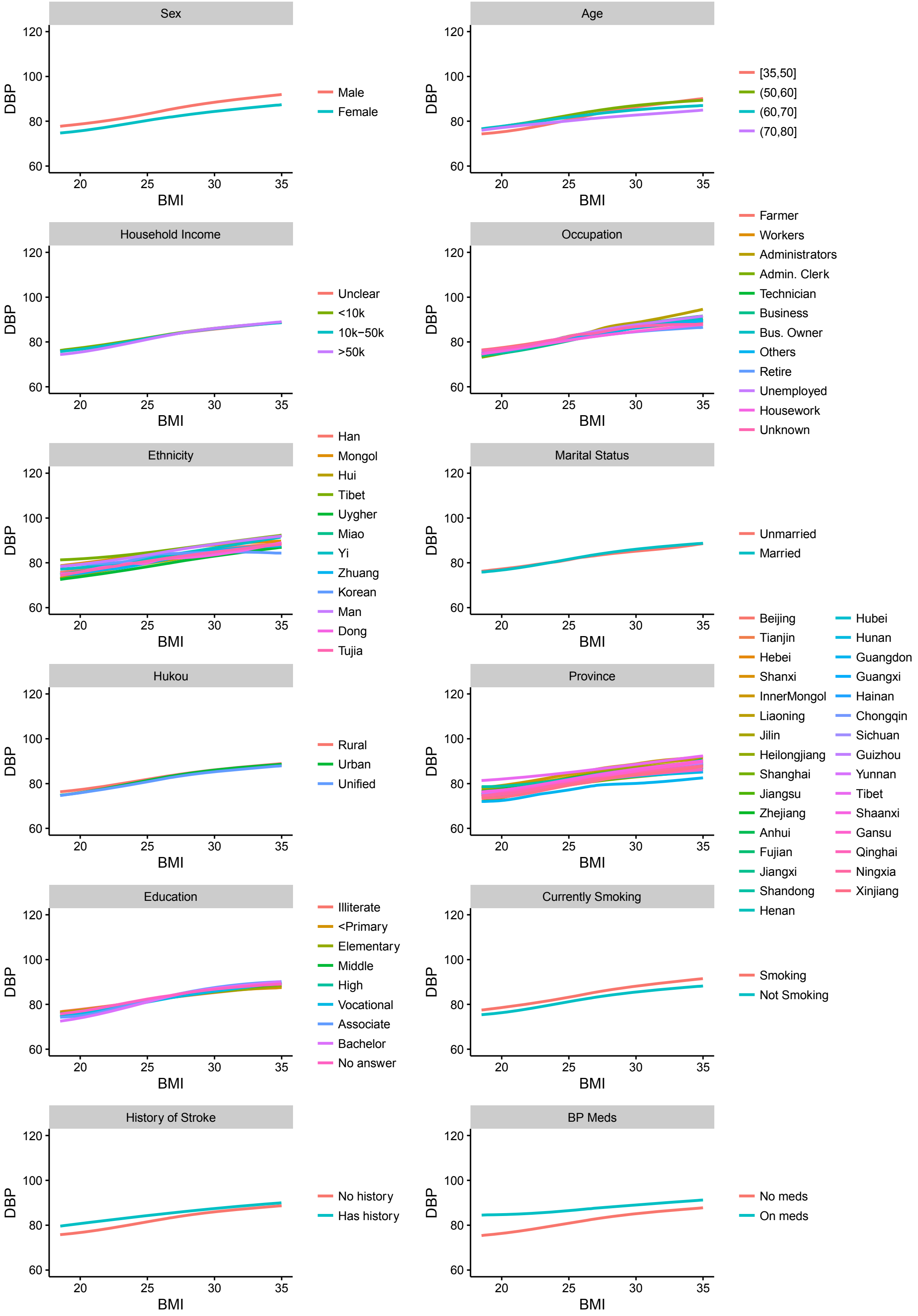

eFigure 3. Histograms and density plots of the Spearman's rank correlation coefficient of body mass index with systolic blood pressure (panels a and b) or with diastolic blood pressure (panels c and d) for every subgroup defined by combinations of covariates

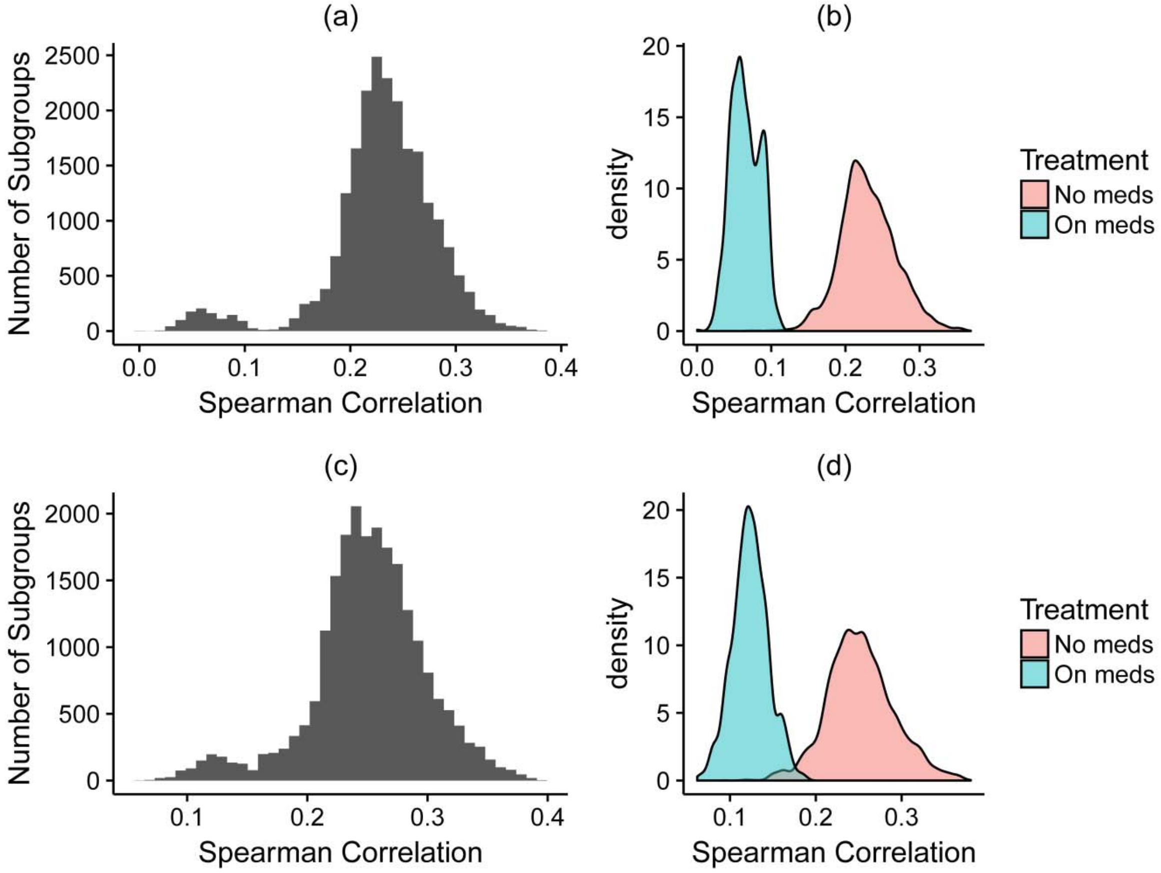

eFigure 4. Distribution of systolic blood pressure (SBP) for individuals on medication before and after matching (panels a and c), and smoothed conditional mean of SBP given body mass index (BMI) before and after matching (panels b and d)

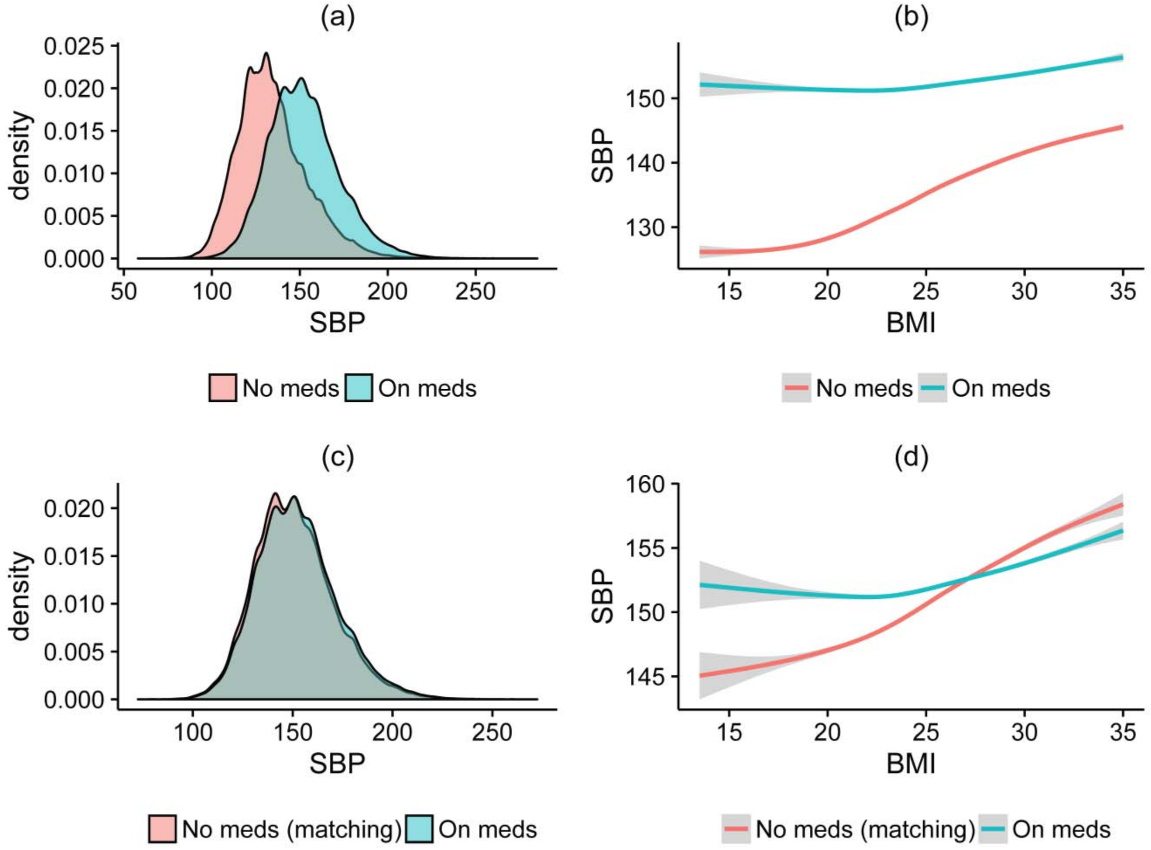

eFigure 5. BMI estimates (red) extended to the year 2030 (blue) using a third order polynomial

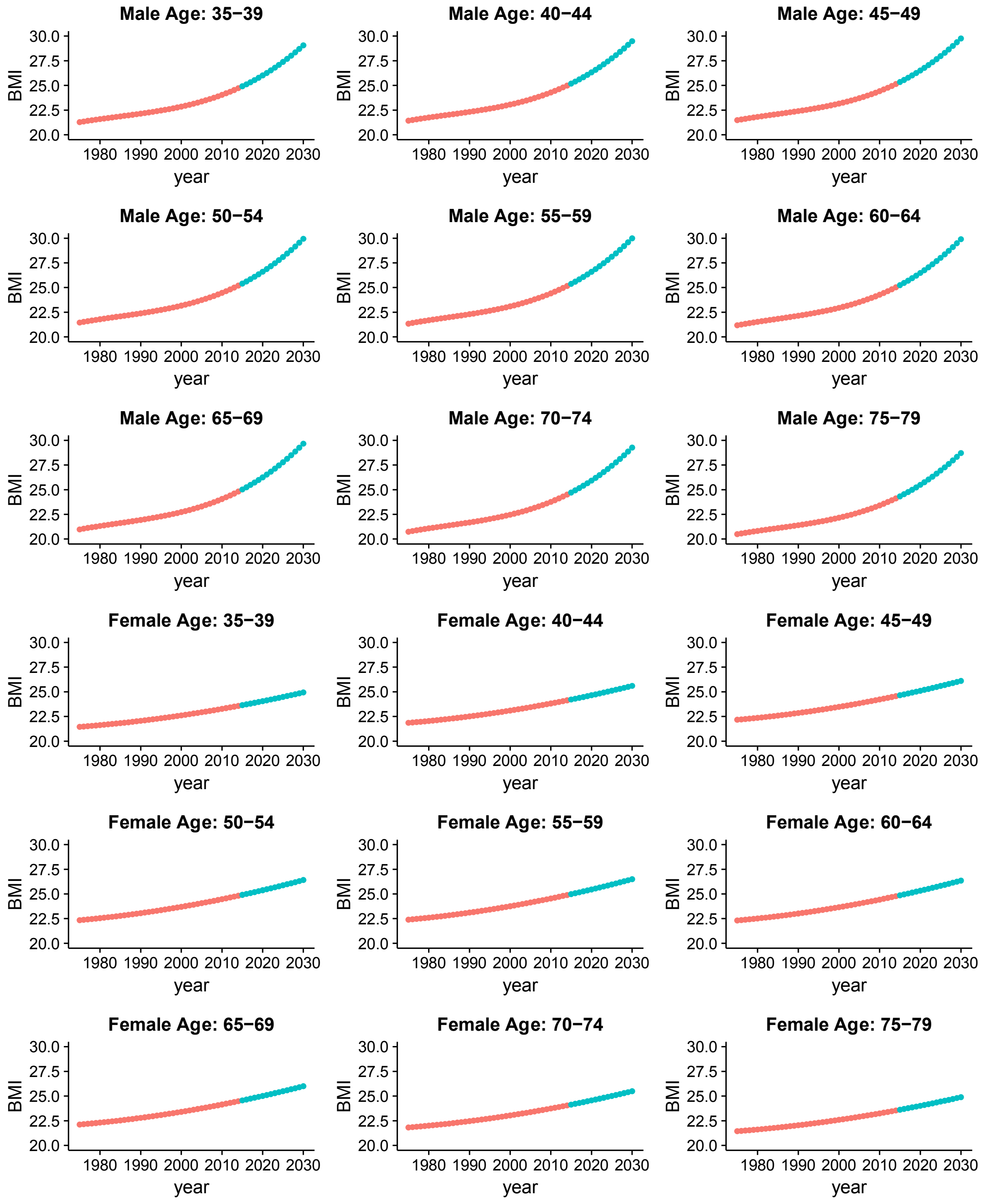

eTable 1. Characteristics of the study population and the increase in blood pressure (mmHg) per 1 kg/m^2 body mass index for each subgroup

Group	N	Mean SBP/DBP (Std. Dev.)	Unadj. SBP Coef.	Adj. SBP Coef.	Unadj. DBP Coef.	Adj. DBP Coef.
**All**						
	1,727,411	137/81 (20/11)	1.37±0.02	1.15±0.02	0.85±0.01	0.75±0.01
**Sex**						
Male	699,700	137/83 (19/11)	1.26±0.03	1.22±0.03	0.94±0.02	0.83±0.02
Female	1,027,711	136/80 (21/11)	1.43±0.02	1.07±0.02	0.79±0.01	0.69±0.01
**Age**						
[35,50]	569,428	129/80 (18/11)	1.52±0.03	1.28±0.03	1.06±0.02	0.88±0.02
(50,60]	549,605	137/82 (20/11)	1.43±0.03	1.17±0.03	0.87±0.02	0.74±0.02
(60,70]	494,061	143/81 (21/11)	1.24±0.03	1±0.03	0.66±0.02	0.6±0.02
(70,80]	114,317	146/80 (21/11)	1.11±0.07	0.87±0.07	0.52±0.04	0.5±0.04
**Household Income**						
Unclear	158,754	136/81 (20/11)	1.3±0.06	1.15±0.05	0.83±0.03	0.75±0.03
<10k	387,718	138/81 (21/12)	1.32±0.04	1.14±0.03	0.8±0.02	0.72±0.02
10k-50k	953,304	136/81 (20/11)	1.38±0.02	1.12±0.02	0.84±0.01	0.73±0.01
>50k	227,635	134/81 (20/11)	1.6±0.05	1.28±0.04	1.02±0.03	0.85±0.03
**Occupation**						
Farmer	841,945	138/81 (21/11)	1.24±0.02	1.08±0.02	0.78±0.01	0.69±0.01
Workers	134,968	132/81 (19/11)	1.61±0.06	1.3±0.06	1.07±0.04	0.88±0.03
Administrators	25,527	131/82 (19/12)	1.73±0.14	1.33±0.14	1.24±0.08	0.93±0.09
Admin. Clerk	31,759	130/81 (19/12)	1.78±0.13	1.32±0.12	1.26±0.07	0.9±0.08
Technician	71,233	130/81 (19/12)	1.78±0.09	1.36±0.08	1.24±0.05	0.94±0.05
Business	43,490	129/80 (19/11)	1.67±0.1	1.37±0.1	1.08±0.06	0.88±0.06
Bus. Owner	34,376	131/81 (19/12)	1.6±0.12	1.31±0.11	1.12±0.07	0.9±0.07
Others	56,734	132/81 (19/11)	1.4±0.09	1.14±0.09	0.9±0.05	0.77±0.05
Retire	288,764	139/81 (20/11)	1.42±0.04	1.09±0.04	0.8±0.02	0.68±0.02
Unemployed	24,148	135/82 (20/12)	1.55±0.14	1.19±0.13	1.04±0.08	0.82±0.08
Housework	142,126	138/81 (21/11)	1.37±0.06	1.11±0.06	0.83±0.03	0.74±0.03
Unknown	26,649	135/82 (20/11)	1.37±0.14	1.09±0.13	0.89±0.08	0.74±0.08

Group	N	Mean SBP/DBP (Std. Dev.)	Unadj. SBP Coef.	Adj. SBP Coef.	Unadj. DBP Coef.	Adj. DBP Coef.
**Ethnicity**						
Han	1,534,086	137/81 (20/11)	1.4±0.02	1.16±0.02	0.87±0.01	0.75±0.01
Mongol	6,807	140/85 (21/12)	1.13±0.27	0.95±0.25	0.74±0.15	0.66±0.14
Hui	10,674	135/80 (21/12)	1.55±0.21	1.21±0.19	0.88±0.12	0.82±0.12
Tibet	26,825	134/84 (23/14)	0.6±0.11	0.58±0.1	0.42±0.07	0.41±0.06
Uygher	14,073	132/79 (22/13)	1.02±0.15	1.08±0.14	0.76±0.08	0.77±0.08
Miao	16,738	135/80 (22/12)	1.15±0.19	1.4±0.18	0.85±0.1	0.9±0.1
Yi	9,707	128/81 (21/12)	1.07±0.23	1.1±0.22	0.84±0.14	0.75±0.14
Zhuang	62,107	134/78 (20/11)	1.53±0.1	1.46±0.09	0.92±0.05	0.82±0.05
Korean	8,531	134/81 (18/10)	1.26±0.26	0.98±0.24	0.68±0.15	0.6±0.14
Man	20,729	141/84 (20/11)	1.58±0.16	1.24±0.15	0.87±0.09	0.76±0.09
Dong	11,256	133/79 (21/10)	1.1±0.24	1.05±0.23	0.77±0.12	0.76±0.12
Tujia	5,878	136/80 (22/12)	1.11±0.32	1.24±0.3	0.85±0.17	0.81±0.17
**Marital Status**						
Unmarried	143,542	139/81 (21/11)	1.27±0.06	1.05±0.06	0.75±0.03	0.67±0.03
Married	1,583,869	136/81 (20/11)	1.38±0.02	1.16±0.02	0.86±0.01	0.76±0.01
**Hukou**						
Rural	951,521	138/81 (21/11)	1.27±0.02	1.1±0.02	0.8±0.01	0.71±0.01
Urban	543,088	135/81 (20/11)	1.6±0.03	1.23±0.03	0.95±0.02	0.81±0.02
Unified	232,625	135/80 (20/11)	1.43±0.05	1.15±0.04	0.9±0.03	0.77±0.03
**Province**						
Beijing	24,747	138/81 (19/11)	1.27±0.13	1.14±0.12	0.74±0.07	0.7±0.07
Tianjin	34,424	137/82 (20/11)	1.58±0.11	1.11±0.1	0.85±0.06	0.69±0.06
Hebei	33,677	139/83 (21/11)	1.29±0.12	1±0.11	0.81±0.06	0.72±0.06
Shanxi	23,641	138/82 (20/11)	1.42±0.14	1.06±0.13	0.77±0.08	0.65±0.08
Inner Mongolia	70,334	141/85 (21/12)	1.33±0.08	1.1±0.08	0.91±0.05	0.79±0.05
Liaoning	132,080	139/83 (20/11)	1.81±0.07	1.32±0.07	0.93±0.04	0.75±0.04
Jilin	134,300	137/82 (19/10)	1.46±0.06	1.2±0.06	0.82±0.03	0.74±0.03
Heilongjiang	25,124	138/82 (21/12)	1.6±0.15	1.33±0.13	0.95±0.08	0.8±0.08
Shanghai	10,639	133/78 (19/11)	1.47±0.21	1.37±0.19	0.9±0.12	0.81±0.11
Jiangsu	83,437	141/82 (21/11)	1.44±0.08	1.08±0.07	0.82±0.04	0.69±0.04
Zhejiang	131,697	139/81 (19/11)	1.33±0.06	1.14±0.06	0.84±0.04	0.75±0.04
Anhui	24,886	137/81 (21/11)	1.2±0.14	1.2±0.13	0.79±0.07	0.79±0.07
Fujian	20,906	134/80 (19/11)	1.3±0.15	1.27±0.14	0.8±0.09	0.8±0.09

Group	N	Mean SBP/DBP (Std. Dev.)	Unadj. SBP Coef.	Adj. SBP Coef.	Unadj. DBP Coef.	Adj. DBP Coef.
Jiangxi	82,483	137/79 (21/11)	1.31±0.09	1.29±0.08	1±0.05	0.93±0.04
Shandong	83,273	141/83 (19/10)	1.03±0.07	0.9±0.07	0.62±0.04	0.58±0.04
Henan	83,686	136/82 (19/11)	1.33±0.07	1.12±0.07	0.94±0.04	0.84±0.04
Hubei	83,039	138/81 (21/11)	1.15±0.07	0.85±0.07	0.75±0.04	0.62±0.04
Hunan	29,855	136/79 (20/11)	1.45±0.13	1.39±0.12	0.8±0.07	0.75±0.07
Guangdong	14,192	130/77 (19/11)	1.3±0.17	1.27±0.16	0.73±0.1	0.74±0.1
Guangxi	137,883	132/78 (20/11)	1.35±0.06	1.26±0.06	0.88±0.03	0.79±0.03
Hainan	6,921	131/78 (20/11)	1.32±0.26	1.34±0.24	0.88±0.15	0.83±0.14
Chongqing	20,592	134/81 (22/12)	1.41±0.18	1.33±0.16	0.93±0.1	0.89±0.09
Sichuan	84,236	136/80 (21/11)	1.16±0.08	1.09±0.08	0.79±0.04	0.75±0.04
Guizhou	73,559	133/80 (22/12)	1.27±0.09	1.34±0.09	0.86±0.05	0.83±0.05
Yunnan	76,392	132/80 (21/12)	1.19±0.09	1.16±0.08	0.88±0.05	0.77±0.05
Tibet	23,582	134/85 (23/14)	0.56±0.12	0.52±0.11	0.4±0.07	0.38±0.07
Shaanxi	83,173	137/81 (21/12)	1.52±0.09	1.23±0.08	0.9±0.05	0.78±0.05
Gansu	10,516	137/80 (22/11)	1.74±0.23	1.54±0.22	0.94±0.12	0.89±0.12
Qinghai	10,556	136/79 (21/12)	1.31±0.24	1.29±0.22	0.76±0.13	0.73±0.13
Ningxia	14,785	136/80 (21/12)	1.6±0.19	1.32±0.18	0.88±0.11	0.8±0.11
Xinjiang	58,796	130/79 (20/12)	1.38±0.08	1.18±0.08	0.86±0.05	0.79±0.05
**Education**						
Illiterate	229,193	141/81 (22/12)	1.18±0.05	0.96±0.04	0.65±0.02	0.61±0.02
<Primary	87,890	140/81 (21/11)	1.07±0.07	0.93±0.07	0.66±0.04	0.6±0.04
Elementary	444,583	138/81 (20/11)	1.26±0.03	1.11±0.03	0.79±0.02	0.71±0.02
Middle	556,445	135/81 (20/11)	1.47±0.03	1.21±0.03	0.91±0.02	0.79±0.02
High	185,240	134/81 (19/11)	1.61±0.05	1.27±0.05	1.03±0.03	0.84±0.03
Vocational	72,796	133/80 (20/11)	1.62±0.09	1.28±0.08	0.98±0.05	0.82±0.05
Associate	77,820	131/81 (19/11)	1.73±0.08	1.3±0.08	1.19±0.05	0.91±0.05
Bachelor	44,668	129/80 (19/12)	1.85±0.11	1.35±0.1	1.29±0.06	0.95±0.07
No answer	26,445	136/82 (20/11)	1.36±0.14	1.1±0.13	0.9±0.08	0.74±0.08
**Currently Smoking**						
Smoking	337,072	136/83 (20/11)	1.17±0.04	1.19±0.04	0.91±0.02	0.79±0.02
Not Smoking	1,390,339	137/81 (21/11)	1.42±0.02	1.13±0.02	0.85±0.01	0.73±0.01
**History of Stroke**						
No history	1,686,749	136/81 (20/11)	1.37±0.02	1.16±0.02	0.85±0.01	0.75±0.01
Has history	40,662	147/84 (21/12)	1±0.12	0.81±0.11	0.65±0.07	0.53±0.06

Group	N	Mean SBP/DBP (Std. Dev.)	Unadj. SBP Coef.	Adj. SBP Coef.	Unadj. DBP Coef.	Adj. DBP Coef.
**BP Meds**						
No meds	1,512,942	134/80 (19/11)	1.24±0.02	1.26±0.02	0.82±0.01	0.8±0.01
On meds	214,469	152/87 (20/12)	0.34±0.05	0.36±0.05	0.43±0.03	0.32±0.03

eTable 2. Predicted increase in body mass index and attributable increase in systolic blood pressure by 2025, population attributable fraction (PAF), and estimate for strokes that can be attributed to the increase in body mass index in men

Men					
Age	BMI Increase	Attributable SBP Increase (mmHg)	PAF	Total Strokes	Attributable Strokes (% of total)
35-39	2·71	3·52	0·22	15943	3559
40-44	2·80	4·17	0·26	88480	22879
45-49	2·88	4·42	0·23	74803	17531
50-54	2·95	4·50	0·24	213320	50788
55-59	3·00	4·20	0·19	217123	40234
60-64	3·03	4·07	0·18	271076	48906
65-69	3·02	4·02	0·14	188521	25673
70-74	2·96	3·55	0·12	243903	29561
75-79	2·86	3·44	0·08	167705	13596
**Women**					
35-39	0·96	1·12	0·08	10374	796
40-44	1·04	1·28	0·09	56682	4932
45-49	1·09	1·52	0·09	48035	4192
50-54	1·12	1·57	0·09	130487	11748
55-59	1·13	1·49	0·07	136740	9542
60-64	1·12	1·40	0·07	212786	13990
65-69	1·07	1·10	0·04	150292	5845
70-74	1·01	1·01	0·04	202091	7203
75-79	0·95	0·99	0·02	153356	3594

eTable 3 Predicted increase in body mass index and attributable increase in systolic blood pressure by 2025, population attributable fraction (PAF), and estimate for ischemic heart disease (IHD) that can be attributed to the increase in body mass index in men

**Men**					
Age	BMI Increase	Attributable SBP Increase	PAF	Total IHD	Attributable IHD (% of total)
35-39	2·71	3·52	0·17	98066	16365
40-44	2·80	4·17	0·19	103291	20084
45-49	2·88	4·42	0·18	117020	20876
50-54	2·95	4·50	0·18	138277	25073
55-59	3·00	4·20	0·14	285950	41262
60-64	3·03	4·07	0·14	360684	50635
65-69	3·02	4·02	0·11	389135	42080
70-74	2·96	3·55	0·1	404935	38894
75-79	2·86	3·44	0·08	399674	30407
**Women**					
35-39	0·96	1·12	0·06	64675	3615
40-44	1·04	1·28	0·06	68614	4359
45-49	1·09	1·52	0·06	90967	5899
50-54	1·12	1·57	0·07	107971	7230
55-59	1·13	1·49	0·05	199416	10660
60-64	1·12	1·40	0·05	268300	13506
65-69	1·07	1·10	0·03	293294	8936
70-74	1·01	1·01	0·03	326865	9107
75-79	0·95	0·99	0·02	390192	8550

eAppendix 1

Projected Rates of Stroke and Ischemic Heart Disease Attributable to Increasing BMI

To estimate the increase in SBP and cardiovascular outcomes associated with increases in BMI, we first predicted the population mean BMI level in China in 2025. We used age-sex- specific mean BMI of China from 1974 to 2014 obtained from the NCD Risk Factor Collaboration[1] and fit a third order polynomial model to predict BMI in 2025. In each subgroup defined by sex and age (categorized into groups of five years), we used linear regression models to predict BP using BMI and 8 covariates: Hukou, marital status, education-level, occupation, household income, smoking, history of stroke, and province. Using the estimates of BMI in 2025 in each group, and all other covariates held constant, we calculated the average increase in SBP attributable to increasing BMI. We then estimated the population attributable fraction (PAF) of strokes and ischemic heart disease (IHD) in 2025 due to the predicted increase in SBP using methods described elsewhere[2-4]. Specifically, we used age- sex-specific relative risk for ischemic stroke[5] and assumed the standard deviation of the SBP in 2025 is the same as the in the current data. We calculated age- sex-specific PAF, and then multiplied PAF by age- sex-specific for incidence of stroke[6] and IHD cases in China in 2013 to calculate the attributable stroke deaths. We estimated the incidence of IHD in China by dividing the number of deaths due to IHD[7] in 2013 by the case fatality rate, as direct estimates of IHD rates in China are not available.

Based on historic age-sex-specific mean BMI in China from 1975-2014, the mean BMI from 2014 to 2025 was predicted to rise from 24·9kg/m^2 to 27·8 kg/m^2 in men and from 24·3 kg/m^2 to 25·3 kg/m^2 in women (eFigure 5). The predicted increase in mean SBP secondary to this increase in mean BMI is 4·0 mmHg in men and 1·3 mmHg in women. In 2025, 20% of strokes in men and 7·3% of strokes in women would be attributable to this increase in SBP. In 2025, roughly 314,569 of the strokes (men: 252,727, women: 61,842) and 357,538 cases of ischemic heart disease (IHD) (men: 285,676, women: 71,862) would be attributable to this increase in SBP (eTables 2 and 3).

eAppendix 2. *Calculation of Population Attributable Fraction*

For a given age group a and sex s, we calculated age-sex-specific population attributable fraction (PAF) using the standard formula[8]

$$PAF_{a,s} = \frac{\int_{l_1}^{l_2} RR_{a,s}P_{a,s}(x)dx - \int_{l_1}^{l_2} RR_{a,s}P_{a,s}{}'(x)dx}{\int_{l_1}^{l_2} RR_{a,s}P_{a,s}(x)dx}.$$

$RR_{a,s}$ is the age-sex specific relative risk of either ischemic stroke or IHD for a unit increase in SBP[5]. $P_{a,s}(x)$ is the population distribution of SBP for the given age and sex group in 2013 estimated as a Gaussian distribution, whereas $P_{a,s}{}'(x)$ is the population distribution of SBP in 2013 after shifting the mean by the projected increase in SBP to approximate the distribution in 2025. l_1, l_2 are the minimum and maximum exposure levels which we took to be 70 and 225 mmHg, as BP outside this range is unlikely to be true. We then multiplied the PAF by age- sex-specific for incidence of stroke in China in 2013 to calculate the attributable stroke incidence[6].

eReferences

1. Collaboration NCDRF. Trends in adult body-mass index in 200 countries from 1975 to 2014: a pooled analysis of 1698 population-based measurement studies with 19.2 million participants. *Lancet*. 2016;387:1377-96.
2. Lim SS, Vos T, Flaxman AD, Danaei G, Shibuya K, Adair-Rohani H, AlMazroa MA, Amann M, Anderson HR and Andrews KG. A comparative risk assessment of burden of disease and injury attributable to 67 risk factors and risk factor clusters in 21 regions, 1990–2010: a systematic analysis for the Global Burden of Disease Study 2010. *The lancet*. 2012;380:2224-2260.
3. Ezzati M, Lopez AD, Rodgers A, Vander Hoorn S, Murray CJ and Group CRAC. Selected major risk factors and global and regional burden of disease. *The Lancet*. 2002;360:1347-1360.
4. Danaei G, Ding EL, Mozaffarian D, Taylor B, Rehm J, Murray CJ and Ezzati M. The preventable causes of death in the United States: comparative risk assessment of dietary, lifestyle, and metabolic risk factors. *PLoS medicine*. 2009;6:e1000058.
5. Singh GM, Danaei G, Farzadfar F, Stevens GA, Woodward M, Wormser D, Kaptoge S, Whitlock G, Qiao Q and Lewington S. The age-specific quantitative effects of metabolic risk factors on cardiovascular diseases and diabetes: a pooled analysis. *PloS one*. 2013;8:e65174.
6. Wang W, Jiang B, Sun H, Ru X, Sun D, Wang L, Wang L, Jiang Y, Li Y and Wang Y. Prevalence, Incidence, and Mortality of Stroke in China. *Circulation*. 2017;135:759-771.
7. Abubakar I, Tillmann T and Banerjee A. Global, regional, and national age-sex specific all-cause and cause-specific mortality for 240 causes of death, 1990-2013: a systematic analysis for the Global Burden of Disease Study 2013. *Lancet*. 2015;385:117-171.
8. Eide GE and Heuch I. Attributable fractions: fundamental concepts and their visualization. *Statistical Methods in Medical Research*. 2001;10:159-193.
